# Aging-Related Changes in Cortical Sources of Sleep Oscillatory Neural Activity Following Motor Learning Reflect Contributions of Cortical Thickness and Pre-sleep Functional Activity

**DOI:** 10.3389/fnagi.2021.787654

**Published:** 2022-01-11

**Authors:** Ahren B. Fitzroy, Bethany J. Jones, Kyle A. Kainec, Jeehye Seo, Rebecca M. C. Spencer

**Affiliations:** ^1^Neuroscience & Behavior Program, University of Massachusetts Amherst, Amherst, MA, United States; ^2^Department of Psychological and Brain Sciences, University of Massachusetts Amherst, Amherst, MA, United States; ^3^Institute for Applied Life Sciences, University of Massachusetts Amherst, Amherst, MA, United States

**Keywords:** aging, sleep, EEG, source estimation, MNE, cortical thickness, motor sequence learning, fMRI

## Abstract

Oscillatory neural activity during sleep, such as that in the delta and sigma bands, is important for motor learning consolidation. This activity is reduced with typical aging, and this reduction may contribute to aging-related declines in motor learning consolidation. Evidence suggests that brain regions involved in motor learning contribute to oscillatory neural activity during subsequent sleep. However, aging-related differences in regional contributions to sleep oscillatory activity following motor learning are unclear. To characterize these differences, we estimated the cortical sources of consolidation-related oscillatory activity using individual anatomical information in young and older adults during non-rapid eye movement sleep after motor learning and analyzed them in light of cortical thickness and pre-sleep functional brain activation. High-density electroencephalogram was recorded from young and older adults during a midday nap, following completion of a functional magnetic resonance imaged serial reaction time task as part of a larger experimental protocol. Sleep delta activity was reduced with age in a left-weighted motor cortical network, including premotor cortex, primary motor cortex, supplementary motor area, and pre-supplementary motor area, as well as non-motor regions in parietal, temporal, occipital, and cingulate cortices. Sleep theta activity was reduced with age in a similar left-weighted motor network, and in non-motor prefrontal and middle cingulate regions. Sleep sigma activity was reduced with age in left primary motor cortex, in a non-motor right-weighted prefrontal-temporal network, and in cingulate regions. Cortical thinning mediated aging-related sigma reductions in lateral orbitofrontal cortex and frontal pole, and partially mediated delta reductions in parahippocampal, fusiform, and lingual gyri. Putamen, caudate, and inferior parietal cortex activation prior to sleep predicted frontal and motor cortical contributions to sleep delta and theta activity in an age-moderated fashion, reflecting negative relationships in young adults and positive or absent relationships in older adults. Overall, these results support the local sleep hypothesis that brain regions active during learning contribute to consolidation-related neural activity during subsequent sleep and demonstrate that sleep oscillatory activity in these regions is reduced with aging.

## Introduction

Sleep is important for consolidating motor sequence learning. Performance improves over a night of sleep or a nap, but not an equivalent interval of wakefulness (Fischer et al., 2002; Walker et al., 2002; Backhaus and Junghanns, 2006; Korman et al., 2007; Doyon et al., 2009b; Debas et al., 2010). Non-rapid eye movement (NREM) sleep features, including oscillatory neural activity in the delta frequency band (0.5–4 Hz; e.g., slow waves, K-complexes), in the theta frequency band (4–8 Hz), and in the sigma frequency band (12–16 Hz; e.g., sleep spindles), have been linked to motor learning consolidation during sleep (Nishida and Walker, 2007; Tucker and Fishbein, 2009; Barakat et al., 2012; Albouy et al., 2013; Tamaki et al., 2013; Fitzroy et al., 2021a).

Consolidation involves synaptic changes in learning-related cortical circuits, and learning induces local, use-dependent changes in sleep oscillatory neural activity apparent in the scalp electroencephalogram (EEG) (“local sleep”; e.g., Huber et al., 2004). Thus, learning-related circuits are expected to contribute to subsequent consolidation-related sleep oscillatory activity. Indeed, source estimation, a method of identifying the neural generators of the scalp-recorded EEG and near-scalp-recorded magnetoencephalogram (MEG), suggests that regions involved in learning contribute to subsequent delta and sigma activity. For example, increased slow wave activity is observed in premotor cortex during NREM sleep following visuomotor learning (Murphy et al., 2011), and increased slow sigma activity is observed in visual cortex during NREM sleep following visual perceptual learning (Bang et al., 2014).

With regard to motor sequence learning, MEG source activity in consolidation-related frequency bands is increased in multiple motor cortical areas during sleep following an explicit sequential finger tapping task, relative to pre-task control sleep (Tamaki et al., 2013). Specifically, motor sequence learning led to increased NREM delta in premotor and primary motor cortices, supplementary motor area (SMA), and pre-SMA; increased NREM theta in primary motor cortex, SMA, and pre-SMA; and increased NREM fast sigma activity in SMA (Tamaki et al., 2013). Moreover, the training-related increases in delta and fast sigma activity in SMA correlated with performance improvements on the motor task over the nap, suggesting that this activity contributed to motor memory consolidation (Tamaki et al., 2013). Together, these results suggest the neural activity during learning prior to sleep has a direct impact on both the set of generators contributing to consolidation-related neural activity during sleep, and the magnitude of activity within these generators.

Aging is associated with reduced consolidation-related sleep oscillatory activity, which may lead to impaired consolidation of motor sequence learning in older adults (e.g., Wilson et al., 2012; Fogel et al., 2014). Delta, theta, and sigma activity measured at the scalp all decline over the adult lifespan (Dijk et al., 1989; Landolt et al., 1996; Carrier et al., 2001; Gaudreau et al., 2001; Nicolas et al., 2001; Crowley et al., 2002; Peters et al., 2014; Schwarz et al., 2017), in a manner that varies topographically across the scalp. Delta declines are broad but largest medial, theta declines are broad but largest medial frontocentral, and sigma declines are focal over only medial frontocentral scalp (Landolt and Borbély, 2001; Münch et al., 2004; Robillard et al., 2010; Carrier et al., 2011; Martin et al., 2013; Mander et al., 2014; Sprecher et al., 2016; Fitzroy et al., 2021b). These decline patterns lead to aging-related increases in the relative frontality of delta, and in the relative laterality of sigma (Sprecher et al., 2016; Fitzroy et al., 2021b).

The topographic variability of aging-related declines in sleep oscillatory activity presumably reflects differential effects of aging on sleep oscillatory activity across brain regions. Aging is also associated with greater fluctuation in the estimated sources of delta during the transition from wake to sleep, further suggesting aging-related differences in the generators of sleep-related neural activity (Tsuno et al., 2002). However, aging-related changes in the estimated sources of sleep oscillatory activity have not been previously investigated. Given that aging-related changes in cortical structure (Mander et al., 2013; Dubé et al., 2015; Varga et al., 2016; Fogel et al., 2017; Latreille et al., 2019; Fitzroy et al., 2021b) and brain activation during learning (Fitzroy et al., 2021a) are associated with changes in the scalp EEG during sleep, it is likely that these factors contribute to changes in the generators of the underlying activity during sleep.

The nature of relationships between aging-related changes in cortical structure and changes in the scalp EEG during sleep suggests changes in the underlying source activity with age. Gray matter atrophy mediates aging-related changes in the magnitude and scalp topography of delta and theta activity in a region-specific manner (Mander et al., 2013; Dubé et al., 2015; Varga et al., 2016; Latreille et al., 2019; Fitzroy et al., 2021b). Gray matter volume also has age-moderated effects on the magnitude and scalp topography of sigma activity in a region-specific manner (Fogel et al., 2017; Fitzroy et al., 2021b). The location of the atrophied brain region and the scalp topography of the influenced sleep oscillatory activity often correspond. For example, gray matter volume loss in frontal medial cortex predicts and mediates declines in delta over frontal scalp (Varga et al., 2016; Fitzroy et al., 2021b), and occipital cortical thinning mediates declines in theta over occipital scalp (Latreille et al., 2019). However, EEG recorded at a specific scalp location is not necessarily a reflection of neural activity under that scalp location. Rather, it is a weighted combination of activity from all generators across the brain (e.g., Luck, 2014). Assessing whether the mediation of aging-related changes in scalp-recorded sleep EEG by gray matter atrophy represents reduced direct contributions of the atrophied regions to sleep oscillatory activity therefore requires estimation of the underlying sources of the scalp EEG. In the present study, we examine the relationship between aging-related differences in estimated cortical sources of scalp-recorded sleep EEG and cortical thickness in the same region to address this question.

In addition to changes in cortical structure, aging-related differences in brain activation during learning are associated with aspects of subsequent sleep EEG, and may therefore drive differences in the underlying source activity. For example, hippocampal activation during motor sequence learning predicts the relative frontality of sigma power during subsequent sleep (Fitzroy et al., 2021a). The rate and magnitude of motor sequence learning is reduced with age, at least under complex and/or explicit task conditions (Curran, 1997; Howard and Howard, 2001; Feeney et al., 2002; Bennett et al., 2007; Rieckmann and Bäckman, 2009), which leads to altered patterns of brain activation during motor sequence learning with age (Aizenstein et al., 2006; Rieckmann et al., 2010; King et al., 2013, 2017b; Fogel et al., 2014; Fitzroy et al., 2021a; but see Daselaar et al., 2003). Aging-related activity reductions are observed in striatal and frontal and motor cortical regions (Aizenstein et al., 2006; Fitzroy et al., 2021a), while aging-related activity increases are sometimes observed in medial temporal and prefrontal areas (Rieckmann et al., 2010; Fogel et al., 2014). Additionally, variability across studies in the brain regions for which gray matter atrophy influences sleep oscillatory activity suggests that task-related brain activity prior to sleep contributes to these relationships. Studies examining sleep after motor sequence learning (Fogel et al., 2017; Fitzroy et al., 2021b) report relationships between gray matter atrophy in motor learning related regions and sleep oscillatory activity that are not observed in studies examining sleep after no specific learning task (Dubé et al., 2015; Latreille et al., 2019). Together, these findings suggest aging-related differences in motor sequence learning lead to differences in source activity during subsequent sleep.

We recently observed relationships between aging-related declines in gray matter volume and changes in the magnitude and topography of sleep delta, theta, and sigma activity at the scalp (Fitzroy et al., 2021b). Additionally, in a subset of these participants, we observed aging-related reductions in striato-cortical network activity during learning of a serial reaction time task (SRTT), along with relationships between brain activity during learning and the topography of sigma activity during subsequent sleep (Fitzroy et al., 2021a). Here, using anatomically informed minimum-norm EEG source estimation and automated cortical thickness measurement in this same cohort, we sought to characterize how aging-related changes in gray matter and pre-sleep learning-related brain activity contributed to aging-related changes in the cortical sources of sleep oscillatory activity.

We hypothesized that the estimated sources of sleep oscillatory activity following motor sequence learning would reflect brain regions active prior to sleep in both young and older adults, and that aging-related declines in estimated source activity would reflect cortical thinning and aging-related differences in brain activity prior to sleep. Specifically, based on prior investigations of estimated source activity in young adults (Tamaki et al., 2013; Brancaccio et al., 2020), we predicted delta, theta, and sigma activity during a midday nap following the SRTT would contain contributions from motor cortical regions in all participants. Additionally, based on known aging-related changes in scalp-recorded sleep EEG magnitude and topography (e.g., Sprecher et al., 2016; Fitzroy et al., 2021b), we predicted source activity would be reduced in older relative to young adults in a region-dependent manner. Moreover, based on prior evidence that gray matter atrophy mediates aging-related declines in scalp-recorded sleep oscillatory activity (Mander et al., 2013; Dubé et al., 2015; Varga et al., 2016; Fogel et al., 2017; Latreille et al., 2019; Fitzroy et al., 2021b), we predicted aging-related thinning in individual cortical regions would mediate aging-related reductions in sleep oscillatory activity within those regions, especially for frontal medial and primary motor cortices. Lastly, based on our prior findings relating brain activation during motor sequence learning to the scalp topography of sleep oscillatory activity in this cohort (Fitzroy et al., 2021a), we predicted aging-related differences in pre-sleep activation of motor sequence learning brain regions would contribute to aging-related differences in source activity during sleep, above and beyond the contributions of cortical thinning.

## Materials and Methods

### Participants

All data were collected as part of a larger study examining relationships between aging-related differences in brain activity during learning and subsequent sleep-dependent consolidation (Fitzroy et al., 2021a,b). In the current analyses, data were included from 17 young adults (8 female) between 18 and 31 years old (*M* = 22.71, *SD* = 3.51), and 18 older adults (8 female) between 58 and 75 years old (*M* = 65.39, *SD* = 5.80). These are the same 35 participants analyzed in Fitzroy et al. (2021a). Participants were excluded during recruitment based on the following criteria: left-handedness; self-reported presence of neurological, psychiatric, cardiac, or sleep disorders; use of medications or supplements affecting sleep; excessive napping, caffeine, or alcohol consumption; habitual bedtime after 12 PM or wake time before 5 AM; and implanted metal or other contraindications for the magnetic resonance imaging (MRI) environment.

All older adults were free of self-reported cognitive decline, and standardized cognitive assessments were administered for 14 of the older adults within the 2 years following participation. Nine older adults completed the Mini-Mental State Exam (MMSE; Folstein et al., 1975), with all scoring above the typical threshold (24; Mitchell, 2009) for detecting cognitive impairment (score range: 27–30). Six older adults completed the Telephone Interview for Cognitive Status (TICS; Brandt et al., 1988), with all scoring within the normal cognitive status range (≥30). As reported in Fitzroy et al. (2021a), young and older adults did not differ on depressive symptoms (Beck Depression Inventory [BDI]; Beck et al., 1996), typical daytime sleepiness (Epworth Sleepiness Scale [ESS]; Johns, 1991), or habitual sleep quality (Pittsburgh Sleep Quality Index [PSQI]; Buysse et al., 1989), but did differ in chronotype (Morningness-Eveningness Questionnaire [MEQ]; Horne and Ostberg, 1976), with older adults typically morning types and young adults typically intermediate types.

### Procedure

All procedures were approved by the Institutional Review Board at the University of Massachusetts Amherst, and written informed consent was obtained from all participants. Participants completed two experimental sessions separated by 1 week (2 weeks for one young adult); sessions were identical other than whether participants were given a nap opportunity or asked to stay awake during a midday interval. Participants were instructed to get quality sleep the night before experimental sessions, and to abstain from caffeine and alcohol consumption the night before and day of experimental sessions.

For each experimental session, participants arrived at the lab at approximately 9 AM, provided informed consent, were screened for MRI contraindications, and were given instructions for the SRTT. Participants then practiced random blocks of the SRTT in a mock MRI scanner, completed the Stanford Sleepiness Scale (SSS; Shahid et al., 2012), were positioned in the real MRI scanner, and underwent a high-resolution T1-weighted structural brain scan. After the T1-weighted scan, participants performed the SRTT while functional MRI (fMRI) images were collected. After completing the morning scan session, participants took a short break including 30 min for lunch in the lab, then had high-density polysomnography (HD-PSG) equipment applied. HD-PSG was recorded during a 2-h nap or wake opportunity (order counterbalanced; order did not affect nap sleep microstructure, see Supplementary Material) from 1 to 3 PM in a private bedroom, which was darkened for the nap opportunity. At 3 PM participants were awakened (nap condition), had the HD-PSG cap and electrodes removed, and were given time to clean up. Participants then performed a second random-only SRTT practice in the mock scanner, completed a second SSS, and returned to the real MRI scanner for a high-resolution diffusion-weighted structural brain scan and to again perform the SRTT during functional imaging. After completing the afternoon scan session, standardized questionnaires were administered to assess sleep habits, sleep quality, chronotype, and self-assessment of task performance.

### Serial Reaction Time Task

Participants performed an explicit variant of the SRTT, in which they were instructed that visual cue sequences would contain a pattern on some blocks and that they should learn that pattern, but not directly informed what the pattern was. Cues were presented on a computer screen positioned behind the scanner bore, made visible to the participant via an angled mirror mounted to the head coil directly in front of their eyes. Movements were cued when one of four horizontally arranged boxes filled white. Participants were instructed to respond to cues quickly and accurately by pressing the button corresponding to the cue location on a four-button MRI-compatible response pad (Current Designs 932). All responses were made using the non-dominant (left) hand. Cues appeared at a regular interval (1000 ms) either randomly with constraint (random blocks), or according to a regular repeating eight-item pattern (sequence blocks) that did not contain repeats, trills, or runs of three or more (3-1-4-2-1-3-2-4 or 2-3-1-2-4-1-3-4; session order counterbalanced across participants), with 40 cues in each block. Participants completed 12 blocks, organized in six pairs of alternating block types (sequence, random), with a 30 s rest period between the third and fourth block-pairs. Block type was indicated on the screen at the start of each block. For full task design details, see Fitzroy et al. (2021a).

### High-Density Polysomnography Acquisition and Preprocessing

HD-PSG data were acquired with reference to FCz during the nap and wake intervals using a custom 129-channel cap (Easycap, Herrsching, Germany) and BrainAmp MR plus amplifiers (Brain Products GmbH, Gilching, Germany). The cap layout consisted of 123 scalp EEG electrodes placed at 10–10 and intermediary locations, four electrooculogram (EOG) electrodes placed beside and below the eyes, and two electromyogram (EMG) electrodes placed over the zygomatic major and mylohyoid muscles. Data were recorded using a hardware bandpass of 0.1–1000 Hz and digitized at 500 Hz using BrainVision Recorder (Brain Products GmbH, Gilching, Germany). Scalp impedances were reduced below 20 kΩ using high-chloride abrasive gel before the nap/wake interval. The wake session was a control condition for our previously reported analyses of sleep-dependent memory consolidation (Fitzroy et al., 2021a), and thus HD-PSG data from the wake session are not analyzed here.

To identify sleep stages, HD-PSG data recorded during the nap interval were offline-filtered (0.3–35 Hz EEG, 10–70 Hz EMG), re-referenced to the contralateral mastoid, and visually scored in 30 s epochs according to American Academy of Sleep Medicine criteria (Iber et al., 2007) using the Hume toolbox (Saletin and Greer, 2015). As originally reported in Fitzroy et al. (2021a), young and older adults did not significantly differ in sleep period time, or in minutes spent in NREM1, NREM2, NREM3, or REM (Supplementary Table 1). Additionally, we calculated minutes of wake after sleep onset (WASO) and a sleep fragmentation index for each participant. Following (Morrell et al., 2012), the sleep fragmentation index was calculated as the total number of shifts from NREM2, NREM3, or REM sleep to either NREM1 or wake over the entire session, divided by the number of hours spent asleep during the session. Young and older adults did not differ in minutes of WASO or in sleep fragmentation (Supplementary Table 1).

To quantify oscillatory neural activity in typical sleep frequency bands at the scalp, the original unfiltered HD-PSG data recorded during the nap were re-referenced to the averaged mastoid, bad channels were visually identified and interpolated, and amplitude envelopes were extracted in the delta (0.5–4 Hz), theta (4–8 Hz), and sigma (12–16 Hz) frequency bands using the filter-Hilbert method (Freeman, 2004). EEG data were filtered using band-specific filter designs: delta using a 2nd order Butterworth IIR filter, theta using a Chebyshev Type II filter requiring ≥20 dB attenuation in the stopband (outside 4–9 Hz) and ≤2 dB attenuation in the passband (4.5–8 Hz), and sigma using a 164th order FIR filter after demeaning the data. Artifactual sections of filtered data were identified automatically for each frequency band and electrode using simple voltage thresholds (delta: ±250 μV; theta/sigma: ±75 μV). Amplitude envelopes were derived at each electrode as the magnitude of the complex analytic signal produced by the Hilbert transform of the bandpass-filtered EEG. Artifact-free regions of the amplitude envelopes recorded during the first 60 min of NREM2 or NREM3 sleep were averaged into non-overlapping 20 s epochs, then averaged again across epochs to give a single amplitude measure per electrode per participant. Scalp EEG preprocessing was completed using a combination of EEGLAB (Delorme and Makeig, 2004), ERPLAB (Lopez-Calderon and Luck, 2014), Fieldtrip (Oostenveld et al., 2011), and in-house MATLAB software (PSGpower^1^).

### MRI Image Acquisition and Preprocessing

Whole-brain images were collected with a Siemens 3T MAGNETOM Skyra scanner (Siemens Healthcare, Erlangen, Germany) and a 20-channel head coil. High-resolution T1-weighted structural images were collected in the sagittal plane using a three-dimensional magnetization-prepared rapid gradient echo (MPRAGE) sequence (TR = 1810 ms, TE = 2.26 ms, TI = 915 ms, FA = 9°, 224 slices, FoV = 224 × 256 × 256 mm^2^, voxel size 0.8 × 0.797 × 0.797 mm^3^). During functional scans, automated scout images were acquired and shimming procedures were performed to optimize field homogeneity, then 372 blood oxygenation level dependent (BOLD) fMRI images were acquired using an interleaved T2*-weighted echo-planar imaging (EPI) sequence (TR = 1500 ms, TE = 30.0 ms, FA = 73°, 60 slices, slice acceleration factor = 3, FoV = 68 × 68 mm^2^, voxel size 3.0 × 3.0 × 2.4 mm^3^).

#### Structural Image Preprocessing

Cortical reconstruction based on the T1-weighted structural images of each individual was performed in FreeSurfer (v6.0.0^2^), using the automated ‘recon-all’ processing stream. Processing included motion correction and averaging (Reuter et al., 2010) of the T1-weighted structural images from the nap and wake sessions, removal of non-brain tissue using a hybrid watershed/surface deformation procedure (Segonne et al., 2004), automated Talairach transformation, segmentation of the subcortical white matter and deep gray matter volumetric structures (Fischl et al., 2002, 2004a), intensity normalization (Sled et al., 1998), tessellation of the gray/white matter boundary, automated topology correction (Fischl et al., 2001; Segonne et al., 2007), and surface deformation following intensity gradients to optimally place the gray/white and gray/cerebrospinal fluid borders at the location where the greatest shift in intensity defines the transition to the other tissue class (Dale and Sereno, 1993; Dale et al., 1999; Fischl and Dale, 2000). The completed cortical models were registered to a spherical atlas based on individual cortical folding patterns to match cortical geometry across subjects (Fischl et al., 1999a,b), and parcellated into regions with respect to gyral and sulcal structure (Fischl et al., 2004b; Desikan et al., 2006). Cortical thickness was calculated as the closest distance from the gray/white boundary to the gray/CSF boundary at each vertex on the tessellated surface (Fischl and Dale, 2000). The procedures for automated measurement of cortical thickness have been validated against manual measurements (Kuperberg et al., 2003; Salat et al., 2004) and histological analysis (Rosas et al., 2002). FreeSurfer morphometric procedures have been demonstrated to show good test-retest reliability across scanner manufacturers and field strengths (Han et al., 2006; Jovicich et al., 2006; Reuter et al., 2012).

#### Functional Image Preprocessing

For full details regarding functional image preprocessing, quantification, and analysis, the reader is referred to Fitzroy et al. (2021a). Briefly, after standard image preprocessing steps, we modeled our random and sequence conditions using individual event-related task regressors for each participant and scan session (pre-nap, post-nap) that maximized the contrast between random and sequence blocks by excluding ambiguous random cues (i.e., trills, runs of three or more, transitional probabilities > 0.33), and were matched in included length to equate signal-to-noise ratio across conditions. We then generated whole-brain images of the sequence > random contrast for each participant and scan session. To reduce dimensionality and facilitate comparisons with source estimate measures, beta weights from the whole-brain contrast images were extracted and averaged separately within seven ROIs using the region of interest extraction (REX) toolbox^3^ : premotor cortex, inferior parietal cortex, hippocampus, parahippocampal gyrus, caudate, putamen, and cerebellum. The premotor cortex and cerebellum ROIs were defined as 10 mm radius spheres, centered on the peak activations of the within-group whole-brain conjunction analysis of activation for sequence vs. random (Fitzroy et al., 2021a). All other ROIs were anatomically defined using the Wake Forest University PickAtlas (Maldjian et al., 2003).

### Source Estimation

The cortical sources of sleep oscillatory activity were estimated separately for each participant and frequency band using depth-weighted minimum-norm estimation (MNE) as implemented in Brainstorm (Hämäläinen and Ilmoniemi, 1994; Tadel et al., 2011, 2019; Gramfort et al., 2014), using individual anatomically accurate forward models based on the cortical reconstructions derived from individual participants’ T1-weighted scans. The anatomically accurate forward models were generated using OpenMEEG (Gramfort et al., 2010), and consisted of three non-intersecting boundary element layers (scalp, outer skull, inner skull) and the cortical surface source space (modeled with 15,002 vertices). Sources of the time-averaged amplitude envelope scalp maps were estimated using dipole orientations constrained to be perpendicular to the cortical sheet (Lin et al., 2006), without modeling noise covariance. The absolute value of each resultant current density map was smoothed using an 8 mm kernel, then projected to standard ICBM152 space using the spherical atlas registration generated during FreeSurfer processing to facilitate across-participant comparisons.

**FIGURE 1:**
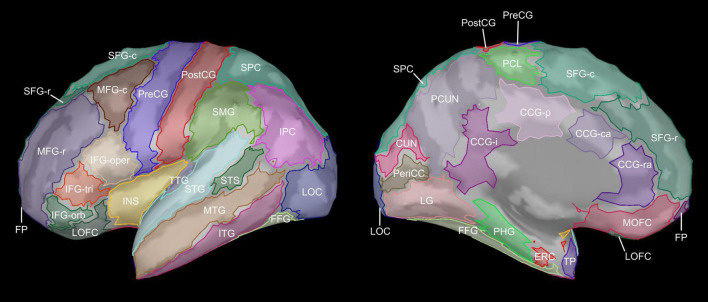
Cortical regions of interest. Cortical regions of interests (ROIs) are shown on the lateral and medial surfaces of the inflated left hemisphere. Bilateral cortical ROIs were defined using the Desikan-Killiany atlas (Desikan et al., 2006) as implemented in FreeSurfer, modified to split the superior frontal gyrus into rostral and caudal subdivisions. CCG-ra, cingulate gyrus (rostral anterior); CCG-ca, cingulate gyrus (caudal anterior); CCG-p, cingulate gyrus (posterior); CCG-i, cingulate gyrus (isthmus); CUN, cuneus; ERC, entorhinal cortex; FFG, fusiform gyrus; FP, frontal pole; IFG-oper, inferior frontal gyrus (pars opercularis); IFG-orb, inferior frontal gyrus (pars orbitalis); IFG-tri, inferior frontal gyrus (pars triangularis); INS, insula; IPC, inferior parietal cortex; ITG, inferior temporal gyrus; LG, lingual gyrus; LOC, lateral occipital cortex; LOFC, lateral orbitofrontal cortex; MFG-r, middle frontal gyrus (rostral); MFG-c, middle frontal gyrus (caudal); MOFC, medial orbitofrontal cortex; MTG, middle temporal gyrus; PCL, paracentral lobule; PCUN, precuneus; PeriCC, pericalcarine cortex; PHG, parahippocampal gyrus; PostCG, postcentral gyrus; PreCG, precentral gyrus; SFG-r, superior frontal gyrus (rostral); SFG-c, superior frontal gyrus (caudal); SMG, supramarginal gyrus; SPC, superior parietal cortex; STG, superior temporal gyrus; STS, superior temporal sulcus (banks); TP, temporal pole; TTG, transverse temporal gyrus.

**FIGURE 2:**
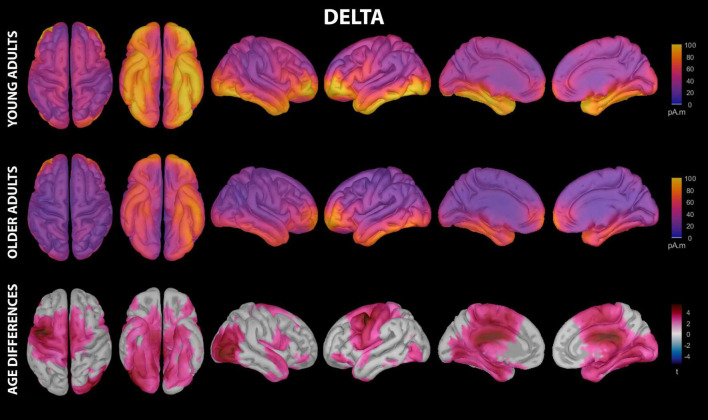
Estimated cortical sources of nap delta activity. Grand average current density maps for the estimated sources of delta activity during the first 60 min of N2/N3 sleep are shown for young adults **(top row)** and older adults **(middle row)**. The results of the vertex-wise permutation test comparing estimated sources of delta activity between young and older adults are shown as the test statistic (*t*) map **(bottom row)**, masked using α = 0.025 to reflect the two-tailed nature of the underlying *t*-tests.

Estimated cortical sources of sleep oscillatory activity were assessed separately for each frequency band and age group using one-tailed one-sample *t*-tests within each ROI of the Desikan-Killiany atlas as implemented in FreeSurfer (Desikan et al., 2006; Figure 1 and Supplementary Table 2), evaluated against the Bonferroni-corrected α = 0.00071. Visual inspection of the full cortex results suggested that rostral and caudal divisions of the superior frontal gyrus contributed differently across frequency bands. To investigate this possibility, and motivated by prior work demonstrating functional subregions of superior frontal gyrus (Li et al., 2013), the superior frontal gyrus ROI was split into rostral and caudal subsections using the ‘subdivide scout’ tool in Brainstorm (Figure 1). Aging-related differences in estimated cortical contributions to sleep oscillatory activity were assessed separately for each frequency band using a Monte Carlo permutation approach (5000 iterations) based on the two-tailed independent-samples *t*-test. The permutation procedure was first performed vertex-wise over the entire cortex (e.g., Figure 2, bottom), with significance evaluated against α = 0.025 to reflect the two-tailed nature of the underlying *t*-tests. Then, to facilitate comparisons with other measures, the permutation procedure was repeated region-wise after averaging estimated source activity within each ROI of the modified Desikan-Killiany atlas. Region-wise statistical significance was evaluated against α = 0.025 while controlling for false discovery rate (FDR) using the Benjamini–Hochberg step-up procedure (Benjamini and Hochberg, 1995), based on the total number of investigated ROIs (70).

### Contributions of Cortical Thinning to Aging-Related Differences in Estimated Source Activity During Sleep

To test the hypothesis that aging-related reductions in cortical activity during sleep are driven by aging-related gray matter atrophy, we performed a set of model-based simple mediation analyses (Imai et al., 2010; Tingley et al., 2014) examining whether aging-related reductions in estimated delta, theta, or sigma source activity within a given cortical region were mediated by aging-related cortical thinning within the same region. We restricted these analyses to only ROIs within which significant aging-related differences in estimated source activity were observed in the frequency band under consideration. For each simple mediation analysis, we estimated the magnitude and reliability of the total effect (TE), average causal mediation effect (ACME), and average direct effect (ADE) using a bootstrap procedure with 1000 iterations. Though it is debated whether a significant TE is a necessary criterion for mediation (Baron and Kenny, 1986; MacKinnon et al., 2000; Agler and De Boeck, 2017), in the interest of conservatism we only assessed potential mediation when a significant TE of age predicting estimated source activity was observed for a given ROI and frequency band. Because we had specific directional hypotheses of positive relationships between cortical thinning and source activity reduction within each ROI, the cortical thinning mediation analyses only considered potential positive relationships, and were therefore evaluated against α = 0.05. For significant mediation results (*p*_*TE*_ < 0.05 and *p*_*ACME*_ < 0.05), the proportion of the TE of age on estimated source activity mediated by cortical thinning was calculated as (ACME + ADE)/ACME.

### Contributions of Pre-sleep Functional Activation to Aging-Related Differences in Estimated Source Activity During Sleep

To test the hypothesis that brain activity prior to sleep influences the set of generators contributing to sleep oscillatory activity, and the magnitude of activity within those generators, we performed a series of regression-based analyses examining the relationships between pre-nap functional activity and estimated source activity during the nap. Specifically, we analyzed relationships between sequence-block-specific pre-nap functional activation within the seven motor sequence learning ROIs examined in Fitzroy et al. (2021a), and estimated source activity within each ROI of the modified Desikan-Killiany atlas for which significant aging-related differences in estimated source activity were observed in the frequency band under consideration. For all regression analyses investigating potential contributions of pre-nap brain activity to estimated source activity during the nap in a given ROI, we included cortical thickness in that same ROI as a covariate to ensure observed effects were not explainable by aging-related differences in cortical structure. Notably, and in contrast to our investigations of the contributions of cortical thickness to estimated source activity, we did not restrict our investigations of the contributions of pre-nap brain activity within a given region to estimated source activity during the nap in only that same region. This is because motor sequence learning follows a progression through multiple subcortical and cortical regions across learning and consolidation (e.g., Doyon et al., 2009a; King et al., 2017a), and it is therefore feasible that pre-sleep activity in one brain region could lead to activity during sleep in another downstream brain region.

For each potential relationship of pre-nap brain activation (PreActivity) to estimated source activity during the nap (EstSource), we first tested whether age moderated the relationship using interaction models of the general form EstSource_*ROI*_ = β0 + β1(CorticalThickness_*ROI*_) + β2(Age) + β3(PreActivity) + β4(Age*PreActivity). When an interaction with age was present, the predictive power of brain activity prior to sleep was assessed separately in young and older adults using the reduced model structure EstSource_*ROI*_ = β0 + β1(CorticalThickness_*ROI*_) + β2(PreActivity). Coefficient statistical significance in the interaction and reduced models was evaluated against α = 0.05 while controlling for FDR within each source ROI using the Benjamini–Hochberg step-up procedure (Benjamini and Hochberg, 1995), to account for multiple pre-nap brain activation predictors.

Second, we tested whether aging-related differences in estimated source activity during the nap were mediated by aging-related changes in pre-nap brain activation using model-based simple mediation analysis, similar to that performed for cortical thinning, while covarying for cortical thickness in the same ROI. Because we did not have specific directional hypotheses regarding relationships between pre-nap functional activation and during-nap estimated source activity, the functional activation mediation analyses considered both potential positive and negative relationships and were therefore evaluated against α = 0.025. For significant mediation results (*p*_*TE*_ < 0.025 and *p*_*ACME*_ < 0.025), the proportion of the TE of age on estimated source activity during the nap mediated by pre-nap functional activation was calculated as (ACME + ADE)/ACME.

## Results

### Delta

Delta activity during the nap had significant contributions from all investigated regions of cortex in both young and older adults, including motor cortical regions (Figure 2 and Supplementary Table 2). Activation peaks were evident in multiple frontal regions (middle frontal gyrus, inferior frontal gyrus, lateral orbitofrontal cortex, frontal pole), temporal regions (entorhinal cortex, parahippocampal gyrus, temporal pole, fusiform gyrus, middle temporal gyrus, inferior temporal gyrus), and lateral occipital cortex in both groups (Figure 2 and Supplementary Table 2).

**FIGURE 3:**
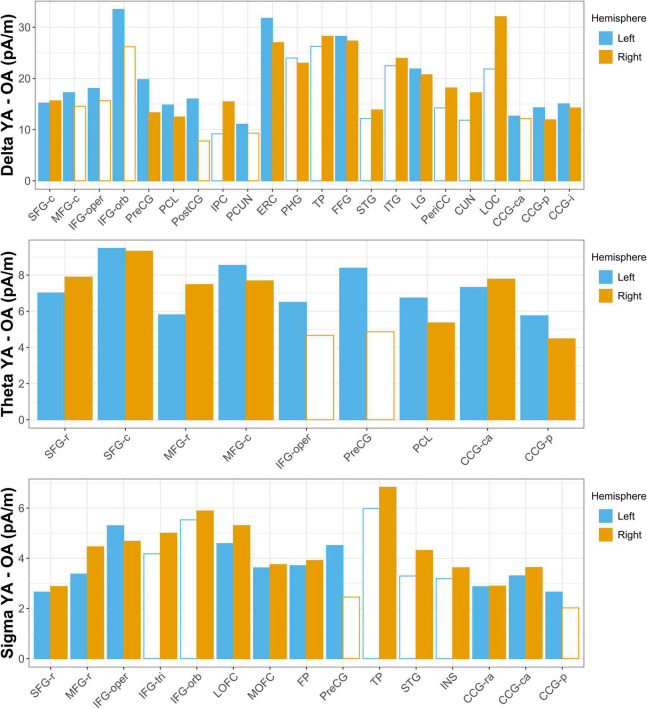
Aging-related differences in estimated source activity. Mean current density differences (young adult [YA] – older adult [OA]) are shown for all ROIs in which the region-wise permutation test revealed a significant effect of age group. Hemispheres in which the aging effect did not reach significance are indicated with empty bars; ROIs for which the aging effect did not reach significance in either hemisphere are omitted for clarity. Abbreviations are the same as those used in Figure 1.

Young adults had increased contributions to delta activity during the nap from several cortical regions compared to older adults, which can be conceptually grouped into four clusters (Figures 2, 3 and Supplementary Table 3): (1) a left-weighted motor cortical network consisting of caudal superior frontal gyrus (i.e., premotor cortex, SMA, pre-SMA), left caudal middle frontal gyrus (i.e., premotor cortex), left inferior frontal gyrus (pars opercularis and pars orbitalis), precentral gyrus (i.e., primary motor cortex), paracentral lobule, left postcentral gyrus, and left precuneus, (2) a right-weighted occipitoparietal network consisting of right inferior parietal cortex, lingual gyrus, right pericalcarine cortex, right cuneus, and right lateral occipital cortex, (3) an assembly of medial and lateral temporal cortical regions consisting of entorhinal cortex, right parahippocampal gyrus, right temporal pole, fusiform gyrus, right superior temporal gyrus, and right inferior temporal gyrus, and (4) cingulate gyrus (left caudal anterior, posterior, and isthmus divisions). No cortical regions were estimated to contribute more to delta during the nap in older adults than in young adults.

### Theta

**FIGURE 4:**
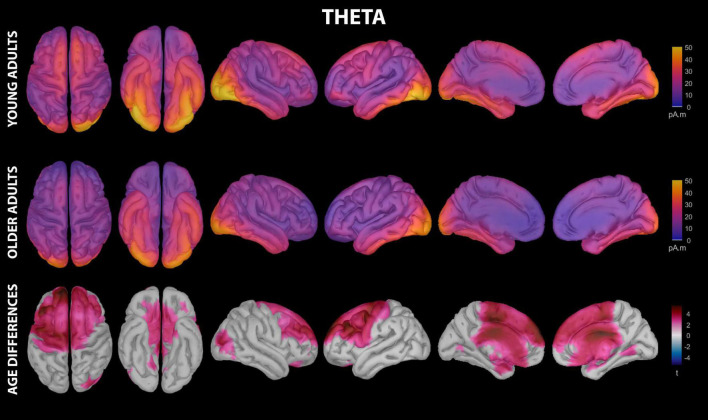
Estimated cortical sources of nap theta activity. Grand average current density maps for the estimated sources of theta activity during the first 60 min of N2/N3 sleep are shown for young adults **(top row)** and older adults **(middle row)**. The results of the vertex-wise permutation test comparing estimated sources of theta activity between young and older adults are shown as the test statistic (*t*) map **(bottom row)**, masked using α = 0.025 to reflect the two-tailed nature of the underlying *t*-tests.

Theta activity during the nap had significant contributions from all investigated regions of cortex in both young and older adults, including motor cortical regions (Figure 4 and Supplementary Table 2). Activation peaks were evident in multiple temporal regions (entorhinal cortex, parahippocampal gyrus, temporal pole, fusiform gyrus, inferior temporal gyrus) and occipital regions (lingual gyrus, pericalcarine cortex, cuneus, lateral occipital cortex) in both groups (Figure 4 and Supplementary Table 2).

Young adults had increased contributions to theta activity during the nap from multiple cortical regions compared to older adults, which can be conceptually grouped into three clusters (Figures 3, 4 and Supplementary Table 4): (1) a left-weighted motor cortical network consisting of caudal superior frontal gyrus, caudal middle frontal gyrus, left inferior frontal gyrus (pars opercularis), left precentral gyrus, and paracentral lobule, (2) a prefrontal network consisting of rostral superior frontal gyrus and rostral middle frontal gyrus, and (3) cingulate gyrus (caudal anterior and posterior divisions). No cortical regions were estimated to contribute more to theta during the nap in older adults than in young adults.

### Sigma

**FIGURE 5:**
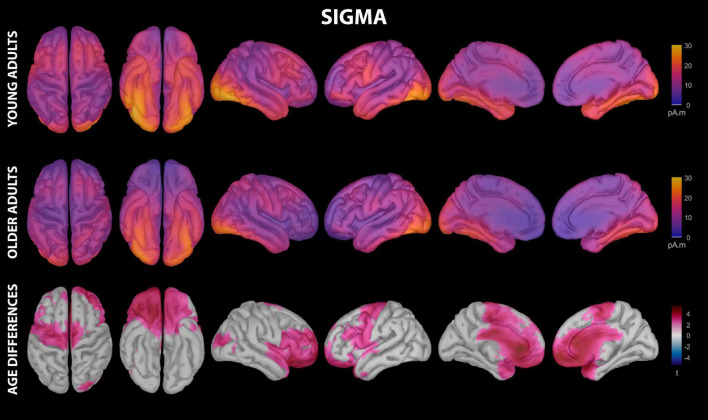
Estimated cortical sources of nap sigma activity. Grand average current density maps for the estimated sources of sigma activity during the first 60 min of N2/N3 sleep are shown for young adults **(top row)** and older adults **(middle row)**. The results of the vertex-wise permutation test comparing estimated sources of sigma activity between young and older adults are shown as the test statistic (*t*) map **(bottom row)**, masked using α = 0.025 to reflect the two-tailed nature of the underlying *t*-tests.

Sigma activity during the nap had significant contributions from all investigated regions of cortex in both young and older adults, including motor cortical regions (Figure 5 and Supplementary Table 2). Activation peaks were evident in multiple temporal regions (entorhinal cortex, parahippocampal gyrus, fusiform gyrus, inferior temporal gyrus) and occipital regions (lingual gyrus, pericalcarine cortex, lateral occipital cortex) in both groups (Figure 5 and Supplementary Table 2).

Young adults had increased contributions to sigma activity during the nap from multiple cortical regions compared to older adults, which can be conceptually grouped into three clusters (Figures 3, 5 and Supplementary Table 5): (1) left precentral gyrus, (2) a right-weighted prefrontal-temporal network consisting of rostral superior frontal gyrus, rostral middle frontal gyrus, inferior frontal gyrus (pars opercularis, right pars triangularis, right pars orbitalis), lateral orbitofrontal cortex, medial orbitofrontal cortex, frontal pole, right temporal pole, right superior temporal gyrus, and right insula, and 3) cingulate gyrus (rostral anterior, caudal anterior, and left posterior divisions). No cortical regions were estimated to contribute more to sigma during the nap in older adults than in young adults.

### Effects of Cortical Thinning

Mean cortical thickness was, on average, lower in older adults than young adults in all investigated regions of cortex (Supplementary Table 2). Moreover, this reduction was largest in frontal regions (Supplementary Table 2), and was significant at the Bonferonni-corrected threshold (α = 0.00071) for all cortical ROIs except an occipital cluster containing lingual gyrus, pericalcarine cortex, lateral occipital cortex, and right cuneus. This cortical thinning mediated aging-related declines in estimated sigma activity within left lateral orbitofrontal cortex and frontal pole, and partially mediated aging-related declines in estimated delta activity in right parahippocampal gyrus, fusiform gyrus, and right lingual gyrus (Table 1).

**TABLE 1:** Mediation of aging-related differences in estimated source activity by same-region cortical thickness.

Lobe	Region of interest	Hem	Mediation							
			*Delta*				*Sigma*			
			ACME (*p*_*ACME*_)	ADE (*p*_*ADE*_)	TE (*p*_*TE*_)	Prop.	ACME (*p*_*ACME*_)	ADE (*p*_*ADE*_)	TE (*p*_*TE*_)	Prop.
Frontal	Lateral orbitofrontal cortex	L					5.75 (<0.001)	–1.16 (0.602)	4.59 (0.004)	1.26
	Frontal pole	L					3.18 (0.036)	0.57 (0.778)	3.75 (0.004)	0.84
		R					3.31 (<0.001)	0.62 (0.642)	3.93 (<0.001)	0.83
Temporal (medial)	Parahippocampal gyrus	R	13.58 (0.050)	9.95 (0.322)	23.53 (0.004)	0.59				
	Fusiform gyrus	L	14.54 (0.048)	13.83 (0.280)	28.37 (0.006)	0.50				
		R	12.50 (0.034)	14.53 (0.134)	27.03 (0.004)	0.45				
Occipital	Lingual gyrus	R	5.10 (0.042)	15.40 (0.016)	20.50 (<0.001)	0.24				

*The magnitude and statistical reliability (p) of the indirect (ACME), direct (ADE), and total effects (TE), and estimated proportion of the total effect accounted for by the indirect effect (Prop.), are shown for all regions in which aging-related declines in mean estimated source activity were significantly mediated (p_ACME_ < 0.05; p_TE_ < 0.05) by cortical thickness averaged within that same region. Age group was dummy coded and centered in the regressions such that young and older adults were coded as positive and negative respectively, meaning that positive ADE and TE values can be interpreted as aging-related declines. Non-significant comparisons are omitted for clarity. Hem, hemisphere; L, left; R, right.*

### Effects of Pre-sleep Functional Activation

Functional activation in the striatum during pre-nap learning related to estimated delta and theta source activity during the nap in multiple frontal and medial cortical regions, in a manner that was moderated by age. Specifically, age moderated the relationships between putamen activation and delta activity in right caudal superior frontal gyrus and left precuneus; between putamen activation and theta activity in caudal superior frontal gyrus, caudal middle frontal gyrus, and cingulate gyrus (caudal anterior and posterior divisions); and between right caudate activation and theta activity in caudal superior frontal gyrus (Table 2). These interactive effects of age and pre-nap striatum activation reflected a general pattern of negative relationships between pre-nap striatum activation and estimated source activity during the nap in young adults, and positive or absent relationships between these measures in older adults (Table 2). Similarly, age moderated the relationship between pre-nap functional activation in left inferior parietal cortex and delta activity during the nap in right caudal superior frontal gyrus, reflecting a negative relationship between these measures in young adults and a marginal positive relationship between these measures in older adults (Table 2). Aging-related differences in estimated source activity during the nap were not mediated by functional activation prior to the nap for any frequency band, source ROI, or functional activation ROI, when covarying for source ROI cortical thickness (*p*s > 0.05).

**TABLE 2:** Significant functional activation regression results.

Functional activation		Estimated source activity			Interaction model		YA model		OA model	
*ROI*	*Hem*	*Band*	*ROI*	*Hem*	*Age x FuncAct*		*FuncAct*		*FuncAct*	
					β	*p*	β	*p*	β	*p*
Putamen	L	Delta	Superior frontal gyrus (caudal)	R	–39.09	**0.004**	–31.82	**0.030**	8.88	0.112
			Precuneus	L	–28.33	**0.003**	–23.58	**0.028**	3.13	0.372
		Theta	Superior frontal gyrus (caudal)	L	–20.74	**0.001**	–16.60	**0.016**	5.61	**0.013**
				R	–23.84	**<0.001**	–17.81	**0.007**	7.66	**0.001**
			Middle frontal gyrus (caudal)	L	–18.15	**0.001**	–12.48	**0.037**	6.12	**0.008**
				R	–23.36	**0.001**	–20.22	**0.003**	4.42	0.208
			Cingulate gyrus (caudal anterior)	L	–14.59	**0.003**	–11.38	**0.033**	3.49	0.055
			Cingulate gyrus (posterior)	L	–10.33	**0.003**	–7.89	**0.035**	2.61	0.104
				R	–10.03	**0.002**	–6.82	**0.039**	3.08	**0.034**
	R	Delta	Superior frontal gyrus (caudal)	R	–34.63	**0.005**	–23.29	**0.035**	11.60	0.067
			Precuneus	L	–24.35	**0.005**	–18.47	**0.019**	4.15	0.307
		Theta	Superior frontal gyrus (caudal)	L	–19.50	**0.001**	–12.29	**0.017**	7.50	**0.002**
				R	–20.85	**<0.001**	–11.86	**0.021**	9.37	**<0.001**
			Middle frontal gyrus (caudal)	L	–18.37	**<0.001**	–10.88	**0.010**	7.45	**0.004**
				R	–17.86	**0.005**	–12.75	**0.015**	5.46	0.176
			Cingulate gyrus (caudal anterior)	L	–15.66	**<0.001**	–11.13	**0.004**	4.76	**0.019**
				R	–14.99	**0.001**	–11.25	**0.007**	4.05	**0.050**
			Cingulate gyrus (posterior)	L	–10.30	**0.001**	–7.07	**0.008**	3.31	0.072
				R	–9.35	**0.001**	–5.53	**0.022**	3.64	**0.029**
Caudate	R	Theta	Superior frontal gyrus (caudal)	L	–12.76	**0.008**	–10.42	**0.032**	3.21	0.104
				R	–14.90	**0.002**	–12.12	**0.010**	4.29	**0.044**
Inferior parietal cortex	L	Delta	Superior frontal gyrus (caudal)	R	–29.22	**0.003**	–21.01	**0.032**	8.14	0.058

*Predictor effect sizes (β) and reliability (p) are reported for all comparisons in which pre-nap functional brain activation (FuncAct) significantly predicted estimated source activity during the nap in an interactive manner with age. When a significant interaction of functional activation and age was observed, the effects of functional activation were examined using follow-up reduced models separately for young adults (YA) and older adults (OA). Cortical thickness from the same ROI as the estimated source activity was included as a predictor of non-interest in the interaction, YA, and OA models. Predictor significance (indicated with bold) was assessed using the Benjamini–Hochberg step-up procedure to hold FDR to 0.05 within each estimated source activity outcome measure. ROI, region of interest; Hem, hemisphere; L, left; R, right.*

## Discussion

Here we demonstrate that sleep oscillatory neural activity following motor sequence learning contains contributions from multiple cortical regions, including those involved in motor sequence learning, in both young and older adults. Further, we demonstrate that these cortical contributions are reduced in older adults in a region-specific manner, and that these aging-related reductions in estimated source activity reflect aging-related changes in both brain structure and pre-sleep brain activation.

### Estimated Cortical Sources of Sleep Oscillatory Activity

Based on prior source estimation work in young adults (Tamaki et al., 2013; Brancaccio et al., 2020) and evidence that motor learning regions are active during the SRTT in young and older adults (e.g., King et al., 2017b), we predicted that sleep oscillatory activity following motor sequence learning would contain contributions from multiple cortical regions, including motor cortices (premotor cortex, primary motor cortex, pre-SMA, SMA), in both young and older adults. Consistent with this prediction, we observed significant estimated contributions to delta, theta, and sigma activity during sleep after motor sequence learning from all regions of the modified Desikan-Killiany atlas, including caudal superior frontal gyrus, caudal middle frontal gyrus, precentral gyrus, and paracentral lobule (which collectively encompass premotor cortex, primary motor cortex, pre-SMA, and SMA; Nieuwenhuys et al., 2008).

Although local activation peaks were evident in these motor cortical regions, especially in young adults, motor cortical regions were not the largest contributors to sleep oscillatory activity following motor sequence learning. Rather, global activation peaks were evident in other cortical regions in a frequency-specific manner. The largest contributions to delta were in prefrontal and ventral temporal regions, whereas the largest contributions to theta and sigma were in ventral temporal and occipital regions. Regarding delta and theta, our pattern of EEG-estimated source activity in a nap after motor sequence learning is markedly similar to that previously reported by Brancaccio et al. (2020), who used similar source estimation techniques on overnight sleep (relative to wake) MEG data after no specific learning task. This converging evidence across overnight MEG and nap EEG data, and across sleep following varied learning task requirements, suggests that the contributions of frontal and temporal regions to sleep delta activity, and of temporal and occipital regions to sleep theta activity, are a basic aspect of sleep physiology not related to sleep pressure, circadian influences, pre-sleep brain activation, or the specific neural populations assessed by the EEG or MEG techniques.

Regarding sigma, our pattern of estimated source activity diverges from that of Brancaccio et al. (2020), who report increased frontal and medial cortical contributions but decreased occipital contributions for sleep sigma relative to wake sigma. We posit this difference reflects the contrast with wake MEG employed by Brancaccio et al. (2020). Delta and theta band activity are substantially increased overall during sleep relative to wake, whereas sigma band activity is more comparable between the sleep and wake states (e.g., Sazgar and Young, 2019). Contrasting against wake will therefore have a larger overall impact on sleep neural activity in the sigma band than in the delta and theta bands, which explains the frequency-dependent pattern of correspondence between our activation maps and those of Brancaccio et al. (2020).

### Aging-Related Changes in the Estimated Cortical Sources of Sleep Oscillatory Activity

Based on prior examinations of aging-related changes in sleep oscillatory activity at the scalp (e.g., Sprecher et al., 2016; Fitzroy et al., 2021b), we predicted that estimated source activity in the delta, theta, and sigma frequency bands during sleep would be reduced in older relative to young adults in a region-dependent manner. Moreover, given prior evidence of aging-related reductions in frontal and motor cortical activation during motor sequence learning (Aizenstein et al., 2006; Fitzroy et al., 2021a), we predicted that aging-related reductions in estimated source activity would be larger in frontal and motor cortical regions. Broadly speaking, our results are in line with these predictions: aging-related reductions in estimated source activity were observed, varied across brain regions, and were evident in motor regions predicted to be active during the SRTT. Additionally, reductions were observed in non-motor regions which also would likely have been active during the SRTT. In this way, our results are consistent with the local sleep hypothesis that neural activity during sleep reflects neural activation prior to sleep. The specific regional distributions of aging-related differences in estimated source activity differed across frequency bands, which we consider separately below.

#### Delta

Aging-related declines in estimated delta source activity were observed in multiple motor cortical regions implicated in the SRTT. Reduced activity was observed in the premotor cortex, primary motor cortex, SMA, and pre-SMA regions previously shown to contribute to motor-learning-related sleep delta activity in young adults (Tamaki et al., 2013), as well as the premotor and SMA regions that show increased sequence-specific activation during SRTT performance in the present cohort (Fitzroy et al., 2021a). Reduced delta activity was also observed in caudal anterior and posterior cingulate regions, which are anatomically interconnected with premotor and primary motor cortices (Beckmann et al., 2009).

Interestingly, while the post-motor-learning sleep delta activity in motor regions reported by Tamaki et al. (2013) was either bilateral or contralateral to the task-performing hand, in the present study aging-related reductions in post-motor-learning delta in motor regions were larger or only present ipsilateral to the task-performing hand. Tamaki et al. (2013) used a within-subjects design, comparing post-motor-learning sleep to control sleep to isolate the areas of largest learning-related increase. Our opposite reduction pattern with regard to hemisphere could then represent a relative preservation in older adults of delta contributions from motor cortical regions in which post-motor-learning activation is highest. Alternatively, these hemisphericity differences may reflect differences between brain activity during motor sequence learning that requires sequence detection and identification like the SRTT, and that which does not like the explicit sequential finger tapping task employed by Tamaki et al. (2013).

Additionally, we observed aging-related delta reductions in several cortical regions which have not been explicitly shown to contribute to delta activity during post-motor-learning sleep, but that could be associated with aspects of performing a visuomotor SRTT. Delta contributions were reduced from medial temporal and parietal regions associated with memory encoding, retrieval, and sleep-dependent consolidation (Nielsen et al., 2005; Cavanna and Trimble, 2006; Aminoff et al., 2013; Navarro Schröder et al., 2015), from occipitoparietal, medial temporal, and lateral temporal regions associated with visual and object processing (Wang et al., 1999; Grill-Spector et al., 2001; Herlin et al., 2021), from parietal regions associated with spatial attention (Numssen et al., 2021), from frontal regions associated with processing rule-governed structure in temporal sequences (Tyler et al., 2011), and from auditory and somatosensory regions, potentially reflecting the loud, uncomfortable MRI scanner environment (Bushnell et al., 1999; Gaab et al., 2006). Given that all of these regions are plausibly associated with SRTT performance, aging-related reductions in delta contributions from them may reflect lower overall activation of task-relevant regions in older adults during the task, and therefore reduced activity in these regions in subsequent sleep. Alternatively, given that ventral frontal and temporal regions have also been implicated in delta generation following no specific learning task (Brancaccio et al., 2020), it is possible that aging-related differences in delta contributions from these regions reflect a general (i.e., not task specific) aging-related reduction in delta activity. Future work investigating aging-related differences in delta activation following different learning tasks is required to separate these interpretations.

#### Theta

Similar to delta, older adults showed reduced estimated theta source activity in a left-weighted cluster of motor cortical regions, which included the primary motor cortex and SMA regions in which Tamaki et al. (2013) reported training-related increases in theta activity, as well as the premotor cortex and pre-SMA regions in which training-related increases did not reach significance in Tamaki et al. (2013). Older adults also showed reduced theta activity in the same cingulate regions that showed aging-related delta reductions, and which are anatomically interconnected with premotor and primary motor cortices.

In further similarity to delta, our age-related reduction in primary motor cortex theta activity was ipsilateral only, whereas that observed by Tamaki et al. (2013) was contralateral only. These theta contributions from additional motor regions relative to Tamaki et al. (2013), and opposite hemisphericity, could again reflect either the within- vs. between-subjects designs of the two studies, or possible differences in theta generators following implicit vs. explicit motor sequence learning. However, despite these differences, both our theta findings and those of Tamaki et al. (2013) are consistent with the hypothesis that cortical regions involved in motor sequence learning contribute to neural activity during subsequent sleep.

Unlike delta, older adults additionally showed large reductions in estimated theta activity in rostral superior frontal and rostral middle frontal gyri. Rostral superior frontal cortex is suggested to work together with the anatomically interconnected left pars opercularis, which also showed reduced theta activity in older adults, in the execution of cognitive manipulations (Li et al., 2013). Similarly, rostral middle frontal cortex is implicated in executive functioning and working memory processes (Michalski et al., 2017). These prefrontal, non-motor cortical regions are likely to have been active during the SRTT. Aging-related reductions in theta contributions from these regions are therefore consistent with lower overall activation of task-relevant regions in older adults prior to the nap, and reduced activity in these regions during subsequent sleep. Moreover, previous investigations of cortical contributions to sleep theta activity following no specific learning task do not show large contributions of rostral superior frontal or rostral middle frontal gyri (Brancaccio et al., 2020), suggesting that the aging-related difference in the present study is related to the SRTT prior to sleep. However, this interpretation remains speculative until aging-related differences in theta generators during sleep following different learning tasks are directly compared.

#### Sigma

Older adults showed reduced estimated sigma source activity in left (ipsilateral) precentral gyrus, corresponding to primary motor cortex, and the anatomically interconnected left posterior cingulate gyrus. This differs from the regions previously shown to contribute to sleep sigma following motor sequence learning in young adults by Tamaki et al. (2013), who report increased fast sigma activity in contralateral SMA and, to a lesser extent, premotor and primary motor cortices.

This variability between studies likely reflects differences in the depth of motor sequence encoding in young adults prior to sleep. In the study by Tamaki et al. (2013), young adults’ performance on the motor sequence task improved over post-training sleep, and improvements correlated with SMA fast sigma activity during sleep. Young adults in the present study, however, were overtrained during the pre-sleep motor sequence task, achieving a cortical motor sequence memory representation and ceiling behavioral performance during the task (Fitzroy et al., 2021a). It is consistent then that sleep sigma activity in SMA correlated with performance improvement in Tamaki et al. (2013), but was not elevated in our young adults, who did not show performance improvement over sleep. However, the aging-related reduction in primary motor cortex sigma activity in the present study suggests that consolidation-related sigma activity did occur during sleep in the young adults, which is consistent with the automatization-related decreases in motor cortex activation during SRTT performance observed over sleep in these young adults (Fitzroy et al., 2021a).

In addition to primary motor cortex, older adults showed reduced sigma activity in a right-weighted cluster of prefrontal and temporal regions implicated in multiple cognitive aspects of the SRTT: cognitive manipulations (Li et al., 2013), attention and inhibition (Japee et al., 2015; Hartwigsen et al., 2019), integrating temporal sequences during decision making (Nogueira et al., 2017), processing higher-order relations (Hartogsveld et al., 2018), and visual object processing (Herlin et al., 2021). Older adults also showed reduced sigma contributions from cingulate regions associated with motor task performance and reward processing (Stevens et al., 2011), and auditory regions. The potential relevance of these non-motor regions’ function to cognitive aspects of the SRTT suggests that the aging-related reductions in sigma activity in these regions may reflect reduced engagement of these regions during the SRTT in older adults, who performed worse on the task (Fitzroy et al., 2021a). However, as with delta and theta activity, confirming this interpretation requires future work comparing aging-related differences in sleep sigma generators following different types of learning. Lastly, older adults showed reduced sigma contributions from right insula, in which reduced gray matter volume has previously been associated with a dominance of fast (vs. slow) spindle activity (Saletin et al., 2013). This reduction likely therefore reflects reduced insular gray matter volume in our older adults (Fitzroy et al., 2021b).

### Cortical Thinning Contributions to Aging-Related Differences in Estimated Source Activity

Based on prior evidence that gray matter atrophy mediates aging-related declines in sleep oscillatory activity at the scalp (Mander et al., 2013; Dubé et al., 2015; Fogel et al., 2017; Latreille et al., 2019; Fitzroy et al., 2021b), we predicted that cortical thinning in individual regions would mediate aging-related reductions in those regions’ contributions to sleep delta, theta, and sigma activity. We additionally predicted that this mediation would be stronger for frontal medial and primary motor cortices, within which gray matter volume loss has been shown to mediate aging-related changes in scalp EEG in a superset of the present cohort (Fitzroy et al., 2021b). Instead, we observed a constrained set of regions within which aging-related reductions in estimated delta and sigma source activity were mediated by same-region cortical thinning. Sigma activity reductions were mediated by cortical thinning in prefrontal regions (left lateral orbitofrontal cortex, frontal pole), whereas delta activity reductions were partially mediated by cortical thinning in medial temporal and occipital regions (right parahippocampal gyrus, fusiform gyrus, right lingual gyrus). The result that aging-related differences in estimated motor cortex source activity were not mediated by cortical thinning suggests that these differences reflect motor cortical activation prior to sleep, rather than aging-related structural brain differences.

Notably, aging-related reductions in estimated frontal medial cortex delta activity were not mediated by cortical thinning, despite prior evidence that frontal medial cortex gray matter loss (volume and thickness) mediates aging-related reductions in scalp-recorded delta activity (Mander et al., 2013; Latreille et al., 2019; Fitzroy et al., 2021b). This result indicates that the mediating role of frontal medial cortex atrophy in aging-related sleep delta reductions at the scalp is not a result of reduced delta activity in frontal medial cortex with aging. Rather, this likely points to the role of frontal medial cortex as a structural and functional network hub (van den Heuvel and Sporns, 2013; Fernández, 2017). From this view, frontal medial cortex plays a coordinating role in delta activity generation, such that atrophy in this region impairs regulation of downstream regions that are the actual delta generators. Additionally, primary motor cortical thickness did not mediate aging-related reductions in primary motor cortical source activity, despite gray matter volume in this region mediating sleep delta and theta reductions at the scalp in a superset of this cohort (Fitzroy et al., 2021b). This result may suggest that the mediation of aging-related scalp delta and theta reductions by primary motor cortex gray matter volume is more driven by gyral surface area than by cortical thickness, as gray matter volume reflects both of these distinct measures, and is particularly influenced by surface area in precentral gyrus (Panizzon et al., 2009; Kong et al., 2015).

### Pre-sleep Functional Activation Contributions to Aging-Related Differences in Estimated Source Activity

We predicted that aging-related differences in pre-sleep activation of motor sequence learning brain regions would contribute to aging-related differences in the estimated sources of oscillatory activity during sleep, above and beyond the contributions of cortical thinning. Consistent with this prediction, we observed a number of age-moderated effects of pre-nap putamen activation on region-specific source activity in the theta, and to a lesser extent delta, frequency bands, while controlling for same-region cortical thickness. We observed similar age-moderated effects of pre-nap caudate activation on superior frontal gyrus theta, and of pre-nap inferior parietal cortex activation on superior frontal gyrus delta.

In all cases, these interactive effects consisted of robust negative relationships in young adults, and positive or absent relationships in older adults. This may reflect differences in the depth of encoding reached in young and older adults prior to sleep. In young adults, ceiling behavioral performance (indicating a high depth of encoding) was achieved during learning, and accompanied by reductions over sleep in SRTT activation of frontal and motor cortical regions. Such reductions are proposed to reflect increases in cortical efficiency (Fitzroy et al., 2021a). Putamen activation increases with practice across the slow phase of motor sequence learning (Hikosaka et al., 1999; Penhune and Steele, 2012; Albouy et al., 2015). The negative relationships between pre-nap putamen activation and cortical source activity during the nap in young adults could therefore reflect higher cortical efficiency during the nap in young adults who progressed farther through slow learning prior to sleep. Conversely in older adults, behavioral improvement was observed over sleep (indicating a low to moderate depth of pre-sleep encoding), and accompanied by increases over sleep in SRTT activation of frontal and motor cortical regions. Increased putamen activation in older adults could therefore reflect further progress through slow learning prior to the nap, and as a result, higher consolidation-related activity in frontal and motor cortical regions during sleep.

Notably, although the restriction of observed aging-related differences in estimated source activity to task-related regions supports the local sleep hypothesis that regions active prior to sleep will be active during sleep, we did not observe any relationships between functional activity in a region prior to the nap and source activity in that same region during the nap. We posit this result reflects a fundamental difference between our fMRI-based and EEG-based neuroimaging approaches. Our fMRI-based measures are necessarily defined as a contrast between two conditions, specifically random and sequence blocks of the SRTT. Low-level aspects of the SRTT, such as visual stimulation and response preparation, were the same between random and sequence blocks, meaning that brain activation related to these aspects is absent from our fMRI contrast. Conversely, our estimated source activity measures do not require an explicit contrast, and therefore reflect local sleep effects due to all aspects of pre-sleep cognitive activity, not just those specific to sequence learning. Thus, while the sequence-specificity of our fMRI contrast is advantageous for understanding during-task activation unto itself, it likely reduces the predictive power of the fMRI-based measures for the source activity measures. Our observation of multiple predictive relationships between pre-sleep putamen activity and cortical activity during sleep then likely reflects a particularly pivotal role of the putamen in aging-related differences in motor sequence learning and consolidation, which is consistent with prior work examining neural activity during post-motor sequence learning sleep with simultaneous fMRI and EEG in young adults (Vahdat et al., 2017).

### Limitations

The regional specificity of the observed aging-related differences in source activity, along with their limited attribution to cortical thinning and the observed influences of some pre-sleep brain activity on source activity, suggests that the aging-related differences in source activity are related to the performance of the SRTT prior to sleep. However, our study design did not include a control sleep condition following either a different learning task, or no specific learning task. Comparing aging-related differences in source activity following motor sequence learning versus other types of learning, or no specific learning task, would give more direct evidence that the aging effects were attributable to pre-sleep motor sequence learning. Our work therefore provides preliminary support for this hypothesis, and should be followed up with work directly comparing aging-related source activity differences during sleep following different learning tasks.

Additionally, in the interest of keeping the overall number of comparisons low, we constrained our investigation of cortical thinning influences to only same-region mediation, and our investigation of pre-sleep brain activity influences to only functional ROIs which we have previously examined in this dataset (Fitzroy et al., 2021a). Similarly, we limited all investigations of influences on aging-related differences in source activity to regions in which the main effect of age was significant. As a result, our methods would have missed second-order or higher effects of cortical thinning on downstream generators of sleep oscillatory activity, potential effects of pre-sleep activation in non-motor cortical regions on sleep oscillatory activity, and crossover interaction effects with age that were unaccompanied by an age main effect. We accept this as a necessary tradeoff to limit the potential for type I error.

Lastly, although we employed state of the art techniques for EEG source estimation, we did not model noise covariance of the EEG. Unlike MEG, it is not possible to collect a room noise sample using EEG equipment disconnected from the participant. Best practices are to therefore use an appropriate baseline in the EEG recording as a model of noise covariance (Tadel et al., 2019). This is difficult for source estimation of sleep EEG however, as there is effectively no *in vivo* baseline for sleep; sleep is already the most quiescent typical brain state. Some studies have used eyes-closed wake EEG recordings as a baseline condition, but this method has conceptual issues. Waking EEG activity in the sigma band includes alpha and beta band activity, which reflects attention and other cognitive factors (e.g., Engel and Fries, 2010) and is variable over time. Subtracting this variable wake activity from the sleep EEG would introduce it as epiphenomenal inverted signals. Future work estimating sleep EEG sources could consider using a bioelectrically appropriate phantom to collect a room noise sample prior to recording, which could be weighted by participant-specific impedance measures to approximate recording noise. Our source estimation techniques were also limited to the cortical surface, despite *a priori* evidence that subcortical structures are involved in motor sequence learning and likely contribute to subsequent sleep neural activity. Future work should investigate the source contributions of deep structures, as analytic techniques for extracting this information from scalp-recorded EEG (e.g., Attal et al., 2009) continue to improve.

## Conclusion

Aging-related reductions in estimated sleep delta, theta, and sigma source activity following motor sequence learning are observed in several motor and other cortical regions associated with aspects of motor sequence learning, in a manner consistent with the local sleep hypothesis (Huber et al., 2004). These aging-related differences are largely unexplained by cortical thinning, which explains aging-related differences in only a small number of cortical generators of delta and sigma activity. Pre-sleep sequence-learning-specific functional activation in the striatum and inferior parietal cortex have age-moderated effects on delta and theta contributions from multiple frontal and motor cortical regions, providing additional evidence that pre-sleep neural activity influences neural activity during sleep. Taken together, these results support the local sleep hypothesis. Moreover, these results identify putamen, caudate, and inferior parietal cortex as brain regions that may be amenable to targeted training paradigms to enhance motor sequence learning and subsequent sleep-dependent consolidation in older adults.

## Data Availability Statement

The raw data supporting the conclusions of this article will be made available by the authors, without undue reservation.

## Ethics Statement

The studies involving human participants were reviewed and approved by the Institutional Review Board at the University of Massachusetts Amherst. The patients/participants provided their written informed consent to participate in this study.

## Author Contributions

AF: conceptualization, methodology, software, formal analysis, investigation, data curation, writing – original draft, and visualization. BJ: conceptualization, methodology, and writing – original draft. KK: investigation, data curation, and writing – review and editing. JS: data curation and writing – review and editing. RS: conceptualization, supervision, project administration, funding acquisition, and writing – review and editing. All authors: contributed to the article and approved the submitted version.

## Conflict of Interest

The authors declare that the research was conducted in the absence of any commercial or financial relationships that could be construed as a potential conflict of interest.

## Publisher’s Note

All claims expressed in this article are solely those of the authors and do not necessarily represent those of their affiliated organizations, or those of the publisher, the editors and the reviewers. Any product that may be evaluated in this article, or claim that may be made by its manufacturer, is not guaranteed or endorsed by the publisher.

## Footnotes

1
https://osf.io/qsryf/

2
http://surfer.nmr.mgh.harvard.edu/

3
http://web.mit.edu/swg/software.htm
