# Supplementary material for: Aging-Related Changes in Cortical Sources of Sleep Oscillatory Neural Activity Following Motor Learning Reflect Contributions of Cortical Thickness and Pre-sleep Functional Activity
Source: Front Aging Neurosci. 2022 Jan 11;13:787654. doi: 10.3389/fnagi.2021.787654 (PMC8786737; doi:10.3389/fnagi.2021.787654)
Supplement: Supplementary file 1 [file Data_Sheet_1.docx]

Supplementary Material

# Supplementary Data

To assess whether the order of the nap and wake experimental sessions could have affected sleep, we analyzed sleep macro- and microstructure (at the scalp and source level) as a function of session order. Session order and age group were fully crossed in our design, with 8 young adults and 9 older adults in the nap/wake (nap-first) group, and 9 young adults and 9 older adults in the wake/nap (wake-first) group. In summary, these analyses showed limited effects of session order on sleep macrostructure that did not interact with age group, and no effects of session order on sleep microstructure at either the scalp or source levels. These analyses and results are described in detail below.

## Potential effects of session order on sleep macrostructure

To analyze potential effects of session order on sleep macrostructure, we performed two-way ANOVAs with between-subjects factors Age (young, older) and Session Order (nap-first, wake-first) separately for total sleep time, minutes spent in NREM1, NREM2, NREM3, REM, and wake after sleep onset (WASO), and sleep fragmentation during the nap. Session Order had no effect on total sleep time (*p*s > 0.6), on minutes spent in NREM2 (*p*s > 0.6), REM (*p*s > 0.6), or WASO (*p*s > 0.1), or on sleep fragmentation (*p*s > 0.06). The nap-first group spent fewer minutes in NREM1 (nap-first: *M* = 9.76, *SD* = 6.86; wake-first: *M* = 18.06, *SD* = 12.87; F(1,31) = 5.30, *p* = 0.028), and more minutes in NREM3 (nap-first: *M* = 21.65, *SD* = 16.51; wake-first: *M* = 10.72, *SD* = 12.74; F(1,31) = 4.70, *p* = 0.038), than the wake-first group. Importantly however, Session Order did not interact with Age for either of these measures (*p*s > 0.4), indicating that influences of Session Order on sleep macrostructure could not explain Age differences in sleep macrostructure.

## Potential effects of session order on sleep microstructure

To analyze potential effects of session order on sleep microstructure at the scalp level, we performed between-subjects (Session Order: nap-first, wake-first) non-parametric cluster-based permutation tests (Maris & Oostenveld, 2007; Oostenveld et al., 2011) over the topography of the time-averaged amplitude envelope scalp maps generated within the delta, theta, and sigma frequency bands (see Section “High-Density Polysomnography Acquisition and Preprocessing” in the main text for details of how the scalp maps were generated). Between-groups independent-samples *t*-values were calculated for amplitude at each electrode, and sufficiently large *t*-values (i.e., those resulting in *p* < 0.025, reflecting the two-tailed test) of the same sign were clustered by adjacency. No minimum number of electrodes was required for clusters. Mass scores were calculated by summing all *t*-values within each cluster, and cluster reliability (*p*-value) was determined empirically using a Monte Carlo simulation with 5000 iterations. Cluster significance was evaluated against α = 0.025, reflecting the two-tailed test. No significant clusters of difference between the nap-first and wake-first groups were found for delta, theta, or sigma activity.

To analyze potential effects of session order on sleep microstructure at the source level, we performed region-wise between-subjects (Session Order: nap-first, wake-first) permutation testing of estimated delta, theta, and sigma source activity averaged within each ROI of the modified Desikan-Killiany atlas. The region-wise permutation testing by Session Order procedure was the same as that described for the region-wise permutation testing by Age in Section “Source Estimation” of the main text. Region-wise statistical significance was evaluated against α = 0.025 while controlling for false discovery rate (FDR) using the Benjamini-Hochberg step-up procedure (Benjamini & Hochberg, 1995), based on the total number of investigated ROIs (70). No significant differences in estimated delta, theta, or sigma activity were observed in any ROI between the nap-first and wake-first groups in the permutation testing.

## References for Supplementary Data

Benjamini, Y., & Hochberg, Y. (1995). Controlling the false discovery rate: A practical and powerful approach to multiple testing. *Journal of the Royal Statistical Society. Series B (Methodological)*, *57*(1), 289–300.

Maris, E., & Oostenveld, R. (2007). Nonparametric statistical testing of EEG- and MEG-data. *Journal of Neuroscience Methods*, *164*(1), 177–190. https://doi.org/10.1016/j.jneumeth.2007.03.024

Oostenveld, R., Fries, P., Maris, E., & Schoffelen, J.-M. (2011). FieldTrip: Open source software for advanced analysis of MEG, EEG, and invasive electrophysiological data. *Computational Intelligence and Neuroscience*. https://doi.org/10.1155/2011/156869

# Supplementary Tables

Supplementary Table 1 – Sleep macrostructure. Group means, group standard deviations, and *p*-values from independent-samples *t*-tests between age groups for sleep macrostructure variables. The sleep fragmentation index was calculated as the total number of shifts from NREM2, NREM3, or REM sleep to either NREM1 or wake over the entire session, divided by the total time spent asleep during the session (i.e., total sleep time) in hours. WASO = wake after sleep onset.

|  | **Young adults** | **Older adults** | **Age differences** |
| --- | --- | --- | --- |
|  | *M (SD)* | *M (SD)* | *p* |
| **Sleep period time (min)** | 112.5 (12.2) | 105.5 (21.7) | 0.25 |
| **Total sleep time (min)** | 98.2 (19.2) | 92.8 (23.2) | 0.45 |
| **NREM1 (min)** | 16.4 (13.5) | 11.8 (8.0) | 0.23 |
| **NREM2 (min)** | 53.8 (16.9) | 58.3 (17.1) | 0.45 |
| **NREM3 (min)** | 17.1 (14.8) | 15.1 (16.5) | 0.71 |
| **REM (min)** | 10.9 (8.0) | 7.7 (11.4) | 0.34 |
| **WASO (min)** | 14.2 (13.9) | 12.8 (9.7) | 0.73 |
| **Sleep fragmentation index** | 5.0 (2.2) | 5.6 (3.2) | 0.51 |

Supplementary Table 2 – Mean cortical ROI measures. Delta, theta, and sigma current density estimates and cortical thickness were averaged within the 35 ROIs in the modified Desikan-Killiany atlas as implemented in FreeSurfer, separately by hemisphere and age group. Mean current density estimates were significantly greater than 0 in all ROIs for all frequency bands in both age groups, evaluated against the Bonferroni-adjusted α = 0.00071 (largest observed *p*-value across all current density estimate tests = 1.01e-10). Aging-related reductions in cortical thickness were significant (α = 0.00071) in all ROIs except for lingual gyrus, pericalcarine cortex, lateral occipital cortex, and right cuneus. Hem = hemisphere; L = left; R = right.

| **Lobe** | **Region of interest** | **Hem** | **Mean current density estimates (pA/m)** | | | | | | **Cortical thickness (mm)** | |
| --- | --- | --- | --- | --- | --- | --- | --- | --- | --- | --- |
|  |  |  | ***Young adults*** | | | ***Older adults*** | | | ***Young adults*** | ***Older adults*** |
|  |  |  | *Delta* | *Theta* | *Sigma* | *Delta* | *Theta* | *Sigma* |  |  |
| Frontal | Superior frontal gyrus (rostral) | L | 37.997 | 16.180 | 9.464 | 38.255 | 9.171 | 6.812 | 2.722 | 2.472 |
|  |  | R | 37.541 | 17.662 | 10.346 | 37.286 | 9.777 | 7.471 | 2.732 | 2.481 |
|  | Superior frontal gyrus (caudal) | L | 40.115 | 25.085 | 12.901 | 24.924 | 15.611 | 9.205 | 2.697 | 2.461 |
|  |  | R | 41.937 | 25.929 | 13.551 | 26.318 | 16.616 | 10.195 | 2.715 | 2.471 |
|  | Middle frontal gyrus (rostral) | L | 58.469 | 17.201 | 11.319 | 51.962 | 11.400 | 7.946 | 2.822 | 2.553 |
|  |  | R | 58.960 | 18.839 | 12.719 | 49.092 | 11.369 | 8.262 | 2.822 | 2.566 |
|  | Middle frontal gyrus (caudal) | L | 42.035 | 22.488 | 14.546 | 24.816 | 13.954 | 10.601 | 2.809 | 2.545 |
|  |  | R | 44.811 | 23.624 | 15.154 | 30.256 | 15.947 | 11.790 | 2.792 | 2.540 |
|  | Inferior frontal gyrus (pars opercularis) | L | 55.261 | 17.955 | 14.563 | 37.217 | 11.464 | 9.264 | 2.845 | 2.579 |
|  |  | R | 51.550 | 16.489 | 13.037 | 35.891 | 11.824 | 8.361 | 2.843 | 2.590 |
|  | Inferior frontal gyrus (pars triangularis) | L | 70.063 | 15.536 | 13.217 | 55.041 | 13.224 | 9.037 | 2.841 | 2.575 |
|  |  | R | 67.304 | 16.612 | 12.924 | 48.934 | 12.445 | 7.923 | 2.847 | 2.593 |
|  | Inferior frontal gyrus (pars orbitalis) | L | 101.075 | 21.842 | 15.825 | 67.593 | 16.108 | 10.294 | 2.804 | 2.551 |
|  |  | R | 85.641 | 20.369 | 14.386 | 59.455 | 14.888 | 8.499 | 2.822 | 2.576 |
|  | Lateral orbitofrontal cortex | L | 70.826 | 17.786 | 12.745 | 53.010 | 13.135 | 8.159 | 2.692 | 2.465 |
|  |  | R | 68.974 | 18.325 | 12.811 | 51.505 | 12.939 | 7.508 | 2.710 | 2.486 |
|  | Medial orbitofrontal cortex | L | 49.538 | 14.758 | 10.192 | 42.340 | 10.201 | 6.572 | 2.522 | 2.320 |
|  |  | R | 45.592 | 13.209 | 9.272 | 41.290 | 8.766 | 5.524 | 2.564 | 2.358 |
|  | Frontal pole | L | 75.220 | 13.060 | 9.033 | 79.324 | 10.916 | 5.326 | 2.717 | 2.475 |
|  |  | R | 73.101 | 13.709 | 9.056 | 80.253 | 11.305 | 5.146 | 2.738 | 2.503 |
|  | Precentral gyrus | L | 45.499 | 21.672 | 13.146 | 25.719 | 13.292 | 8.636 | 2.708 | 2.475 |
|  |  | R | 41.755 | 19.693 | 12.239 | 28.452 | 14.829 | 9.784 | 2.702 | 2.471 |
|  | Paracentral lobule | L | 37.719 | 19.146 | 10.231 | 22.901 | 12.416 | 7.676 | 2.494 | 2.302 |
|  |  | R | 35.084 | 17.856 | 9.994 | 22.617 | 12.502 | 8.041 | 2.502 | 2.301 |
| Parietal | Postcentral gyrus | L | 41.827 | 19.090 | 11.859 | 25.833 | 13.606 | 9.438 | 2.643 | 2.422 |
|  |  | R | 35.453 | 16.862 | 10.629 | 27.676 | 14.662 | 10.675 | 2.643 | 2.419 |
|  | Supramarginal gyrus | L | 34.806 | 13.584 | 10.554 | 25.929 | 14.282 | 10.902 | 2.741 | 2.516 |
|  |  | R | 31.264 | 13.923 | 9.936 | 26.226 | 14.009 | 11.159 | 2.765 | 2.531 |
|  | Superior parietal cortex | L | 39.057 | 19.590 | 11.588 | 27.035 | 18.774 | 12.188 | 2.451 | 2.269 |
|  |  | R | 39.834 | 22.799 | 11.787 | 28.505 | 18.606 | 12.483 | 2.453 | 2.278 |
|  | Inferior parietal cortex | L | 36.691 | 19.051 | 11.078 | 27.521 | 20.771 | 12.895 | 2.627 | 2.445 |
|  |  | R | 39.949 | 23.361 | 11.464 | 24.517 | 17.427 | 10.433 | 2.659 | 2.475 |
|  | Precuneus | L | 35.451 | 15.535 | 10.325 | 24.414 | 14.073 | 10.426 | 2.326 | 2.159 |
|  |  | R | 33.141 | 15.711 | 10.087 | 23.836 | 13.795 | 10.105 | 2.326 | 2.171 |
| Temporal (medial) | Entorhinal cortex | L | 92.893 | 30.006 | 18.594 | 61.158 | 22.345 | 13.870 | 2.765 | 2.596 |
|  |  | R | 86.373 | 28.535 | 18.547 | 59.382 | 22.968 | 14.505 | 2.750 | 2.583 |
|  | Parahippocampal gyrus | L | 92.701 | 30.070 | 17.578 | 68.714 | 26.792 | 16.937 | 2.541 | 2.404 |
|  |  | R | 83.022 | 29.307 | 19.608 | 60.039 | 24.831 | 17.555 | 2.486 | 2.352 |
|  | Temporal pole | L | 93.521 | 27.593 | 18.515 | 67.247 | 20.915 | 12.532 | 2.903 | 2.715 |
|  |  | R | 94.004 | 26.667 | 18.650 | 65.777 | 21.116 | 11.822 | 2.878 | 2.693 |
|  | Fusiform gyrus | L | 94.088 | 35.243 | 19.482 | 65.869 | 28.772 | 17.675 | 2.655 | 2.521 |
|  |  | R | 83.151 | 34.005 | 21.418 | 55.844 | 27.178 | 17.720 | 2.667 | 2.542 |
| Temporal (lateral) | Superior temporal gyrus | L | 50.518 | 15.608 | 12.753 | 38.366 | 15.438 | 9.459 | 2.945 | 2.729 |
|  |  | R | 55.112 | 17.027 | 13.476 | 41.270 | 16.228 | 9.163 | 2.971 | 2.755 |
|  | Middle temporal gyrus | L | 63.972 | 21.441 | 13.500 | 52.286 | 22.578 | 11.544 | 2.951 | 2.764 |
|  |  | R | 61.950 | 22.413 | 14.958 | 49.449 | 21.525 | 11.385 | 2.955 | 2.775 |
|  | Inferior temporal gyrus | L | 89.202 | 31.758 | 17.197 | 66.725 | 28.160 | 15.211 | 2.877 | 2.715 |
|  |  | R | 81.815 | 31.874 | 20.135 | 57.886 | 27.047 | 15.727 | 2.890 | 2.734 |
|  | Transverse temporal gyrus | L | 42.666 | 13.216 | 10.519 | 31.496 | 13.059 | 8.289 | 2.921 | 2.692 |
|  |  | R | 41.546 | 12.817 | 10.337 | 32.075 | 12.621 | 7.744 | 2.952 | 2.719 |
|  | Superior temporal sulcus (banks) | L | 38.428 | 13.313 | 8.966 | 34.862 | 16.670 | 9.639 | 2.874 | 2.668 |
|  |  | R | 36.262 | 14.246 | 9.752 | 31.461 | 14.475 | 8.009 | 2.906 | 2.696 |
|  | Insula | L | 46.188 | 14.261 | 10.913 | 33.671 | 11.694 | 7.725 | 2.820 | 2.589 |
|  |  | R | 50.934 | 15.394 | 11.322 | 37.392 | 12.543 | 7.697 | 2.800 | 2.573 |
| Occipital | Lingual gyrus | L | 63.603 | 29.805 | 17.197 | 41.752 | 24.282 | 16.232 | 2.230 | 2.142 |
|  |  | R | 56.967 | 27.765 | 16.817 | 36.245 | 21.822 | 15.728 | 2.240 | 2.167 |
|  | Pericalcarine cortex | L | 48.647 | 26.765 | 15.160 | 34.409 | 25.317 | 15.545 | 2.167 | 2.073 |
|  |  | R | 48.456 | 27.895 | 16.312 | 30.314 | 23.502 | 14.294 | 2.172 | 2.111 |
|  | Cuneus | L | 38.826 | 22.433 | 12.854 | 26.986 | 22.131 | 13.546 | 2.208 | 2.091 |
|  |  | R | 42.882 | 26.243 | 14.315 | 25.683 | 22.101 | 12.641 | 2.202 | 2.119 |
|  | Lateral occipital cortex | L | 73.300 | 37.835 | 20.056 | 51.457 | 36.343 | 19.223 | 2.389 | 2.285 |
|  |  | R | 73.604 | 44.004 | 22.454 | 41.541 | 32.138 | 16.931 | 2.414 | 2.326 |
| Cingulate | Cingulate gyrus (rostral anterior) | L | 39.985 | 11.076 | 7.563 | 40.452 | 7.085 | 4.695 | 2.510 | 2.303 |
|  |  | R | 45.517 | 11.492 | 7.620 | 42.799 | 7.364 | 4.728 | 2.527 | 2.316 |
|  | Cingulate gyrus (caudal anterior) | L | 36.971 | 19.450 | 11.417 | 24.340 | 12.137 | 8.116 | 2.450 | 2.251 |
|  |  | R | 37.817 | 19.076 | 11.321 | 25.681 | 11.306 | 7.688 | 2.471 | 2.263 |
|  | Cingulate gyrus (posterior) | L | 36.882 | 18.603 | 11.627 | 22.615 | 12.856 | 8.974 | 2.325 | 2.157 |
|  |  | R | 34.535 | 17.295 | 11.297 | 22.607 | 12.821 | 9.273 | 2.338 | 2.160 |
|  | Cingulate gyrus (isthmus) | L | 41.891 | 16.757 | 11.862 | 26.856 | 13.498 | 10.893 | 2.192 | 2.054 |
|  |  | R | 38.667 | 16.034 | 11.486 | 24.447 | 12.543 | 9.986 | 2.187 | 2.052 |

Supplementary Table 3 – Region-wise aging-related differences in delta source activity. Only significant results are shown, evaluated against α = 0.025 to reflect the two-tailed test while controlling for FDR based on the number of investigated ROIs (70).

| **Delta (0.5 – 4.0 Hz)** | | | | | |
| --- | --- | --- | --- | --- | --- |
| **Lobe** | **Region of interest** | **Left hemisphere** | | **Right hemisphere** | |
|  |  | *t* | *p* | *t* | *p* |
| Frontal | Superior frontal gyrus (caudal) | 3.347 | 0.002 | 3.038 | 0.004 |
|  | Middle frontal gyrus (caudal) | 3.910 | <0.001 | – | – |
|  | Inferior frontal gyrus (pars opercularis) | 3.035 | 0.005 | – | – |
|  | Inferior frontal gyrus (pars orbitalis) | 2.721 | 0.010 | – | – |
|  | Precentral gyrus | 4.250 | <0.001 | 3.106 | 0.005 |
|  | Paracentral lobule | 3.535 | 0.001 | 3.518 | 0.001 |
| Parietal | Postcentral gyrus | 3.382 | 0.001 | – | – |
|  | Inferior parietal cortex | – | – | 3.464 | 0.001 |
|  | Precuneus | 2.888 | 0.009 | – | – |
| Temporal (medial) | Entorhinal cortex | 3.485 | 0.002 | 3.084 | 0.004 |
|  | Parahippocampal gyrus | – | – | 2.779 | 0.010 |
|  | Temporal pole | – | – | 2.854 | 0.009 |
|  | Fusiform gyrus | 3.006 | 0.008 | 3.344 | 0.003 |
| Temporal (lateral) | Superior temporal gyrus | – | – | 2.921 | 0.006 |
|  | Inferior temporal gyrus | – | – | 2.963 | 0.006 |
| Occipital | Lingual gyrus | 3.177 | 0.005 | 3.384 | 0.002 |
|  | Pericalcarine cortex | – | – | 3.193 | 0.001 |
|  | Cuneus | – | – | 3.151 | 0.003 |
|  | Lateral occipital cortex | – | – | 4.896 | <0.001 |
| Cingulate | Cingulate gyrus (caudal anterior) | 2.818 | 0.009 | – | – |
|  | Cingulate gyrus (posterior) | 4.057 | <0.001 | 3.506 | 0.001 |
|  | Cingulate gyrus (isthmus) | 3.513 | 0.002 | 3.786 | 0.001 |

Supplementary Table 4 – Region-wise aging-related differences in theta source activity. Only significant results are shown, evaluated against α = 0.025 to reflect the two-tailed test while controlling for FDR based on the number of investigated ROIs (70).

| **Theta (4.0 – 8.0 Hz)** | | | | | | |
| --- | --- | --- | --- | --- | --- | --- |
| **Lobe** | **Region of interest** | **Left hemisphere** | | **Right hemisphere** | | |
|  |  | *t* | *p* | *t* | *p* |  |
| Frontal | Superior frontal gyrus (rostral) | 4.277 | <0.001 | 4.181 | <0.001 |  |
|  | Superior frontal gyrus (caudal) | 3.912 | <0.001 | 3.741 | 0.001 |  |
|  | Middle frontal gyrus (rostral) | 3.385 | 0.001 | 3.717 | <0.001 |  |
|  | Middle frontal gyrus (caudal) | 4.069 | <0.001 | 2.899 | 0.004 |  |
|  | Inferior frontal gyrus (pars opercularis) | 3.266 | 0.004 | – | – |  |
|  | Precentral gyrus | 4.078 | <0.001 | – | – |  |
|  | Paracentral lobule | 3.385 | 0.001 | 3.018 | 0.005 |  |
| Cingulate | Cingulate gyrus (caudal anterior) | 3.833 | <0.001 | 3.877 | <0.001 |  |
|  | Cingulate gyrus (posterior) | 4.364 | <0.001 | 3.713 | <0.001 |  |

Supplementary Table 5 – Region-wise aging-related differences in sigma source activity. Only significant results are shown, evaluated against α = 0.025 to reflect the two-tailed test while controlling for FDR based on the number of investigated ROIs (70).

| **Sigma (12.0 – 16.0 Hz)** | | | | | |
| --- | --- | --- | --- | --- | --- |
| **Lobe** | **Region of interest** | **Left hemisphere** | | **Right hemisphere** | |
|  |  | *t* | *p* | *t* | *p* |
| Frontal | Superior frontal gyrus (rostral) | 2.634 | 0.007 | 2.906 | 0.006 |
|  | Middle frontal gyrus (rostral) | 2.874 | 0.006 | 3.494 | 0.002 |
|  | Inferior frontal gyrus (pars opercularis) | 2.907 | 0.007 | 3.142 | 0.004 |
|  | Inferior frontal gyrus (pars triangularis) | – | – | 3.162 | 0.003 |
|  | Inferior frontal gyrus (pars orbitalis) | – | – | 3.417 | 0.001 |
|  | Lateral orbitofrontal cortex | 2.989 | 0.006 | 3.762 | 0.001 |
|  | Medial orbitofrontal cortex | 3.456 | 0.002 | 3.987 | 0.001 |
|  | Frontal pole | 2.947 | 0.002 | 4.008 | 0.001 |
|  | Precentral gyrus | 3.358 | 0.002 | – | – |
| Temporal (medial) | Temporal pole | – | – | 3.339 | 0.001 |
| Temporal (lateral) | Superior temporal gyrus | – | – | 2.982 | 0.005 |
|  | Insula | – | – | 3.489 | 0.001 |
| Cingulate | Cingulate gyrus (rostral anterior) | 4.011 | <0.001 | 3.859 | 0.001 |
|  | Cingulate gyrus (caudal anterior) | 2.920 | 0.003 | 3.600 | 0.001 |
|  | Cingulate gyrus (posterior) | 3.132 | 0.003 | – | – |
